# Mitochondria and Mood: Mitochondrial Dysfunction as a Key Player in the Manifestation of Depression

**DOI:** 10.3389/fnins.2018.00386

**Published:** 2018-06-06

**Authors:** Josh Allen, Raquel Romay-Tallon, Kyle J. Brymer, Hector J. Caruncho, Lisa E. Kalynchuk

**Affiliations:** ^1^Division of Medical Sciences, University of Victoria, Victoria, BC, Canada; ^2^Department of Psychology, University of Saskatchewan, Saskatoon, SK, Canada

**Keywords:** depression, behavior, reelin, mitochondria, oxidative phosphorylation, antidepressants

## Abstract

Human and animal studies suggest an intriguing link between mitochondrial diseases and depression. Although depression has historically been linked to alterations in monoaminergic pharmacology and adult hippocampal neurogenesis, new data increasingly implicate broader forms of dampened plasticity, including plasticity within the cell. Mitochondria are the cellular powerhouse of eukaryotic cells, and they also regulate brain function through oxidative stress and apoptosis. In this paper, we make the case that mitochondrial dysfunction could play an important role in the pathophysiology of depression. Alterations in mitochondrial functions such as oxidative phosphorylation (OXPHOS) and membrane polarity, which increase oxidative stress and apoptosis, may precede the development of depressive symptoms. However, the data in relation to antidepressant drug effects are contradictory: some studies reveal they have no effect on mitochondrial function or even potentiate dysfunction, whereas other studies show more beneficial effects. Overall, the data suggest an intriguing link between mitochondrial function and depression that warrants further investigation. Mitochondria could be targeted in the development of novel antidepressant drugs, and specific forms of mitochondrial dysfunction could be identified as biomarkers to personalize treatment and aid in early diagnosis by differentiating between disorders with overlapping symptoms.

## Mitochondria

Mitochondria are the main energy factories of eukaryotic cells. The brain is particularly dependent on mitochondrial activity due to both its high levels of energy use and its inability to store large amounts of energy reserves in the form of glycogen. As a result of the their roles in energy production, mitochondria also generate reactive oxygen species (ROS) that may have a toxic effects in cells. In addition, mitochondria also play a prominent role in the regulation of apoptotic cell death (for examples, see Davidson and Hardison, 1984; Herrmann and Neupert, 2000; Calabrese et al., 2001; Chan, 2006; Chipuk et al., 2006; Fattal et al., 2006; McBride et al., 2006; Youle and van der Bliek, 2012; Tobe, 2013; Bansal and Kuhad, 2016).

**FIGURE 1:**
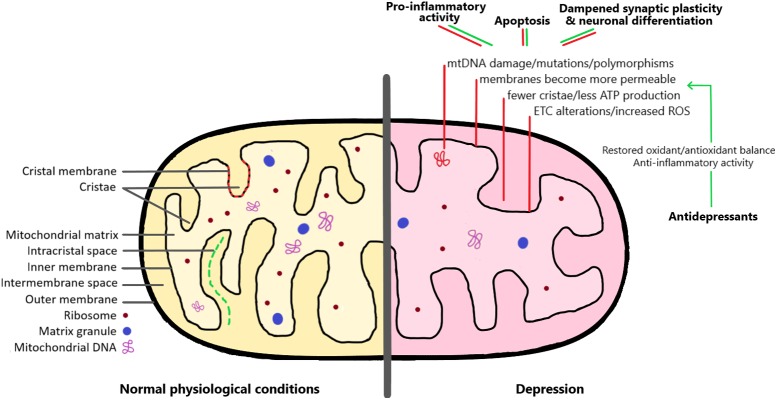
The mitochondrion under normal physiological conditions and in the depression brain. As detailed in the right side of the image, there are a series of mitochondrial alterations that have been observed both in depressed patients and in animal models of depression (red lines). These include changes affecting mitochondrial DNA, membrane permeability, and increased formation of reactive oxygen species (ROS). As a consequence, these alterations lead to pro-inflammatory activity, increased apoptosis, and dampened synaptic plasticity and neuronal differentiation. Interestingly, antidepressant medication can restore the mitochondrial oxidant/antioxidant balance, and therefore help to rescue the negative effects of mitochondrial dysregulation (green lines). See the text for more detailed explanations.

The focus of this review is the link between mitochondrial dysfunction and major depression. Depression has historically been considered a disorder of altered pharmacology and altered hippocampal neurogenesis. However, recent evidence has opened the door to an expanded notion of the neurobiology of depression, such that a reduction in ATP levels, enhancement of oxidative stress, and acceleration of apoptosis are now considered to be important events (reviewed in Rezin et al., 2008a). In this review, we summarize some of the latest knowledge on mitochondrial dysregulation in major depression (depicted in **Figure 1**) and also discuss how mitochondrial dysfunction could instigate downstream changes in extracellular matrix proteins such as reelin, neuronal nitric oxide (nNOS), oxidative stress, and inflammation, and finally adult hippocampal neurogenesis. Uncovering how all these factors influence one another could lead to new vistas in the development of novel therapeutics for the treatment of this problematic disorder.

## Hypotheses About the Neurobiological Basis of Depression

Depression is a common neuropsychiatric disorder, affecting up to 20% of the population (Kessler et al., 1994). The presence and severity of symptoms vary among individuals, and can include low mood and anhedonia, decreased energy, altered appetite and weight, irritability, sleep disturbances, and cognitive deficits (Nemeroff, 1998). Patients with depression have a higher rate of other physical illnesses (i.e., comorbidities with cardiovascular disorders, stroke, etc.), decreased social functioning, and a high mortality rate (Nemeroff, 1998). The complexity of this disorder is further compounded by the fact that it often co-occurs with other psychiatric conditions. For example, about 50% of depression patients also suffer from anxiety disorders (Kessler et al., 1996), which can indicate a more severe form of the disease, with delayed recovery, increased risk of relapse, greater disability, and increased suicide attempts (Hirschfeld, 2001). It is generally thought that a combination of environmental and genetic factors influences the development of depression (Nestler et al., 2002; Kalia, 2005). However, despite extensive clinical and preclinical research efforts, there is still a fundamental lack of understanding about the specific biological changes that give rise to depressive symptoms.

For more than 50 years, the dominant theory for the pathogenesis of depression was the monoamine hypothesis (Schildkraut, 1965), which arose from observations that antidepressant drugs work by inhibiting the reuptake of monoamines such as serotonin and norepinephrine. However, this theory has largely fallen out of favor due to a number of discrepancies, such as the fact that the therapeutic effects of antidepressants take weeks to develop even though monoamine levels are elevated within hours of administration, and the fact that only about 40% of patients respond satisfactorily to treatment (Trivedi et al., 2006). More recent theories about the neurobiological basis of depression have focused on the neurogenesis theory, which posits that stress-induced decreases in hippocampal neurogenesis could be a causal factor in depression (Jacobs et al., 2000). This hypothesis is supported by observations of decreased hippocampal volume in patients with depression (MacQueen et al., 2003; Campbell et al., 2004), decreased cell proliferation and survival in preclinical animal models of depression (Brummelte and Galea, 2010; Schoenfeld and Cameron, 2015), and increased cell proliferation and survival after antidepressant treatment (Santarelli et al., 2003; Fenton et al., 2015). Our laboratory has contributed to this literature using a well-validated preclinical model of depression in which rats or mice are subjected to daily injections of the stress hormone corticosterone (CORT) for several weeks, followed by behavioral testing and then tissue collection for further analyses (Gregus et al., 2005; Johnson et al., 2006; Sterner and Kalynchuk, 2010). Using this approach, we showed that the time course for the onset of depression-like behavior in rats is paralleled by dampened hippocampal neurogenesis and neuronal maturation (Lussier et al., 2013). Importantly, this work also implicated the extracellular matrix protein reelin in the pathogenesis of depression (see Caruncho et al., 2016). There is evidence that reelin can regulate adult hippocampal neurogenesis and dendritic spine plasticity (Pujadas et al., 2010), and our data show that rats subjected to repeated CORT injections have dampened reelin expression selectively in the proliferative subgranular zone of the dentate gyrus (Lussier et al., 2009) and also that antidepressant treatment normalizes depression-like behavior, hippocampal neurogenesis, and hippocampal reelin expression in tandem (Fenton et al., 2015). The reelin link is relevant to mitochondrial dysfunction because reelin in the periphery interacts with the immune system and a loss of reelin can magnify markers of inflammation that influence mitochondria (Green-Johnson et al., 1995). This issue is discussed in more detail in the section below on reelin and inflammation.

It is important to note that the neurogenesis hypothesis of depression is somewhat controversial because depression-like symptoms can occur even when cell proliferation is not decreased and the behavioral actions of antidepressants do not always coincide with increases in the number of hippocampal neurons (Surget et al., 2008; Bessa et al., 2009; David et al., 2009). Instead, some data suggest that depression and the therapeutic actions of antidepressants may be more related to alterations in dendritic complexity and neuronal remodeling than cell number *per se* (Bessa et al., 2009; Lussier et al., 2013). The neurogenesis hypothesis of depression may therefore be better described as the neuroplasticity hypothesis of depression – a broadening of concept that would include plasticity within the cell, such as mitochondrial activity.

Because the brain has high aerobic activity, requiring about 20 times more energy than the rest of the body by weight (Kety, 1950), it is highly vulnerable to conditions stemming from impaired energy production. A resting cortical neuron consumes 4.7 billion ATP molecules every second (Zhu et al., 2012). Evidence from post-mortem (Kato et al., 1997; Konradi et al., 2004; Iwamoto et al., 2005; Munakata et al., 2005), imaging (Frey et al., 2007), genetic (Kato et al., 1997; Kato and Kato, 2000; Iwamoto et al., 2005; Benes et al., 2006), and cellular (Cataldo et al., 2010) studies is plentiful in showing the involvement of mitochondrial dysfunction in bipolar disorder and schizophrenia. Studies are also emerging providing evidence that mitochondria-mediated mechanisms are related to depressive symptoms (reviewed in Castren, 2005; Tobe, 2013; Shimamoto and Rappeneau, 2017; Petschner et al., 2018), that mitochondrial mutations are witnessed in individuals diagnosed with depression (Munakata et al., 2007; Ben-Shachar and Karry, 2008) and that the two diseases are often comorbid (Koene et al., 2009; Morava et al., 2010).

Mitochondria could play a role in the dampened plasticity associated with depression. Depression is associated with abnormalities in intracellular second messenger signal transduction cascades resulting from 5HT and NE receptor activation (Perez et al., 2000; Popoli et al., 2000) and dysregulated and desensitized monoamine receptors (Hamon and Blier, 2013). These observations can be related to mitochondrial dysfunction because ATP is needed for the activation of downstream signaling following the binding of neurotransmitters to receptors (Moretti et al., 2003). ATP is also necessary to attend to the energy demands of vesicle transport and neurotransmitter release (reviewed in Vos et al., 2010; and more recently in Devine and Kittler, 2018). Furthermore, patients with mitochondrial diseases or mitochondrial DNA (mtDNA) mutations and polymorphisms often present symptoms characteristic of mood disorders (Suomalainen et al., 1992; Onishi et al., 1997; Kato et al., 2001; Fattal et al., 2006; Morava et al., 2010). Higher rates of mitochondrial biogenesis are needed for neuronal differentiation (Calingasan et al., 2008) and therefore, dysfunctional mitochondria could result in impaired neuroplasticity in depressed patients.

## Genetics

There have been many observations of links between mtDNA and depression. As mentioned above, depression is a heterogeneous disorder, with several different symptom profiles, and genetic background contributes to its development (Lesch, 2004). Prevalence rates for depression are as high as 54% in patients with mitochondrial diseases (Fattal et al., 2007). However, not all patients who have the same mitochondrial gene mutations develop depressive symptoms, indicating a genetic and non-genetic interplay of factors (Koene et al., 2009). Mitochondrial disorders may result from mutations in nuclear DNA or mtDNA, with the amount of mtDNA mutations possibly correlating with disease severity (Chinnery et al., 2000). In one interesting study, long-PCR revealed that 68% of patients with depression have mtDNA deletions, compared to 36% of control subjects (Gardner et al., 2003). Similarly, in leukocytes of depressed patients, mtDNA copy number variates were significantly lower than in control subjects, and mtDNA oxidative damage was increased (Chang et al., 2015). Interestingly, oxidized mtDNA activates pro-inflammatory cytokines (Adzic et al., 2016) and increased inflammation is known to play a role in the development of depressive symptoms (e.g., Brymer et al., 2018; Wang et al., 2018). Variations in mtDNA have also been shown to cause cognitive impairments in mice (Sharpley et al., 2012) and in humans (Inczedy-Farkas et al., 2014; Petschner et al., 2018), and cognitive deficits are a common symptom associated with depression.

Beyond these general linkages between mtDNA and depression, recent research has implicated a number of specific mitochondrial genes in depression. This topic has been reviewed in detail recently (Petschner et al., 2018), so only a few examples will be mentioned here. A recent systematic assessment using mitochondrial PCR array profiling identified 16 genes that were differentially expressed in the dorsolateral prefrontal cortex of post-mortem brains from depressed patients compared to control subjects (Wang and Dwivedi, 2017). The identified genes are ones known to govern oxidative stress and neuronal ATP levels, suggesting for the first time that mitochondrial genes might be altered in tissue samples from human patients. Similarly, the mitochondrial ATP6V1B2 gene has been implicated in depression, possibly through its effects on neurotransmission and receptor-mediated endocytosis (Petschner et al., 2018). Less direct evidence comes from observations that mice with mutations of the POLG gene, which encodes a subunit of mtDNA polymerase, exhibit depression-like symptoms (Kasahara et al., 2006), and that polymorphisms of genes that code for mitochondrial enzymes, such as MTHFD1L, are associated with negative rumination, which is a precursor to depression (Eszlari et al., 2016). This polymorphism is also associated with high levels of homocysteine, which has been related to hippocampal volume and depression (Moustafa et al., 2014).

These examples point to an intriguing link between mtDNA or gene expression and depression, though more work needs to be done in this area, particularly to identify gene alterations in tissue from human patients.

## Proteomic Studies

There have been many studies indicating the involvement of the oxidative phosphorylation (OXPHOS) pathway in depression. Proteomic studies conducted on post-mortem brains from depressed patients suggest that about 21% of dysregulated proteins are also commonly dysregulated in patients with schizophrenia and bipolar disorder (Saia-Cereda et al., 2017; Villa et al., 2017). In a mutant mouse model of depression, a dysregulated OXPHOS pathway was seen in the hippocampus (Zubenko et al., 2014), which is a key region of dampened plasticity in both human depression and rodent models (Sheline et al., 2003; Frodl et al., 2006; Sterner and Kalynchuk, 2010). In addition, proteomic studies using different animal models of depression have revealed alterations in specific proteins involved in OXPHOS and also confirmed the effect of antidepressant treatment in the expression of these proteins (reviewed in Carboni, 2015).

In depressed patients, most of the differentially expressed proteins are involved in cellular assembly, organization, function, and maintenance, as well as cardiovascular system development and function, but they are mainly related to deregulation of energy metabolism pathways (Martins-de-Souza et al., 2012). Twenty different subunits of the OXPHOS complex were increased in post-mortem brains from depressed patients (Martins-de-Souza et al., 2012), whereas the opposite effect has been seen in brains from patients with schizophrenia (Martins-de-Souza et al., 2011). A proteomics approach also revealed that the SSRI fluoxetine upregulated and downregulated 23 and 60 cytosolic mitochondrial-related proteins, respectively (Filipović et al., 2017). In addition, 60 non-synaptic mitochondrial-related proteins were upregulated whereas three were downregulated. These effects were largely confirmed in a subsequent study (Głombik et al., 2017). When looking at samples from the dorsolateral prefrontal cortex, which shows reduced activation and volume in patients with depression (Drevets et al., 1998; Halari et al., 2009), a shotgun label-free approach revealed that 32% of differentially expressed proteins associated with depression were involved in metabolic/energy pathways (Martins-de-Souza et al., 2012). Two other proteomic studies showed that several proteins involved in energy metabolism, such as carbonic anhydrase and aldolase C, were increased in the frontal cortex (Johnston-Wilson et al., 2000) and anterior cingulate cortex (Beasley et al., 2006) of depressed patients. These results are consistent with PET findings of a reduction in cerebral glucose metabolism in the brains of depressed patients (Baxter et al., 1989), which was reversed by 6 weeks of treatment with the SSRI paroxetine (Kennedy et al., 2001). Similarly, PET studies also revealed that depressed patients had reduced blood flow and bioenergetic metabolism in the prefrontal cortex (Drevets et al., 1997; Mayberg et al., 1999; Moretti et al., 2003), cingulate gyrus, and basal ganglia (Videbech, 2000).

Psychiatric disorders almost always have overlapping symptoms, which might reflect common mechanisms when compared against controls. An interesting study addressing this issue showed that patients with major depression that included psychosis had more differentially expressed proteins associated with energy metabolism, whereas patients with depression without psychosis had changes in proteins associated with cell growth and maintenance, although 53.7% of the altered proteins overlapped (Martins-de-Souza et al., 2012). Subtle differences in proteome fingerprints may become useful biomarkers that could be used to stratify patients with different symptoms profiles and to formulate effective personalized treatment plans. Proteomic studies support the view that mitochondrial dysfunction is one of many important factors involved in depression, and may identify novel pathogenic mechanisms of psychiatric disorders.

That alterations in mitochondria bioenergetics pathways contributed to the pathophysiology of depression also raise the possibility of developing mitochondrial biomarkers that can illustrate a better therapeutic approach to the treatment of depression. However, this field is still undeveloped and additional studies are needed characterizing specific mitochondrial dysfunctions in depression in relation to therapeutic response to antidepressants, or to evaluate the possibility of identifying mitochondrial drug targets that could be used to develop novel antidepressant drugs (Klinedinst and Regenold, 2015).

## Decreased ATP Production

The production of ATP through OXPHOS is a key method by which mitochondria provide energy to the cell. Several lines of research have confirmed that depression is associated with lower than normal levels of ATP production. For example, brain levels of ATP are generally lower in the brains of depressed patients compared to control subjects (Moretti et al., 2003; Martins-de-Souza et al., 2012). This may be related to the dampened neuronal plasticity and impaired hippocampal neurogenesis thought to be operative in depression (Caruncho et al., 2016), as neurogenesis is a metabolically demanding process. Other researchers have found changes in ATP in depression in areas outside the brain. Gardner et al. (2003) found that mitochondrial ATP production rates and mitochondrial enzyme ratios in electron transport chain (ETC) complexes I–IV were significantly decreased in the muscles of patients with depression compared to controls. A correlation was found between altered biochemistry and depression rating scale scores further evidencing the relationship between mitochondrial dysfunction and psychopathology. In peripheral blood mononuclear cells, ATP turnover-related respiration was lowered in depressed patients compared to age-matched controls, as well as routine and uncoupled respiration and coupling efficiency (Karabatsiakis et al., 2014). Moreover, ATP-binding cassette transporters, which utilize the energy of ATP, are also altered in depressed patients and single nucleotide polymorphisms in the gene that codes for these transporters may be indicators of severity of the disorder and patient responsiveness to antidepressants (Lin et al., 2011).

Decreased ATP production has also been observed in preclinical animal models of depression. Female rats displaying anhedonia (i.e., decreased preference for sucrose) after 40 days of mild stress also had decreased hippocampal NA+, K+-ATPase activity (Gamaro et al., 2003). Fluoxetine reversed the effects of stress on enzymatic activity suggesting that NA+, K+-ATPase activity may be involved in the depression-like phenotype. In another study, fluoxetine restored sucrose preference, and normalized ATP synthesis rate and mitochondrial respiratory control in the raphe nucleus after 18 days of chronic unpredictable stress (Wen et al., 2014). Another study using the chronic mild stress paradigm revealed that mice with decreased sucrose preference and increased immobility in the tail suspension test (i.e., learned helplessness) also showed damaged mitochondrial ultrastructure, impaired respiration rates, and altered membrane potentials in the hippocampus, hypothalamus, and the cortex (Gong et al., 2011).

## Oxidative Stress

Mitochondria are the primary source of ROS, which under normal conditions play important roles in cell signaling and homeostasis. ROS are produced in the OXPHOS pathway; however, in normal physiological conditions, mitochondria create protective factors that can neutralize harmful free radicals (Petschner et al., 2018). For example, there is a mitochondrial matrix thiol system that has an important role in antioxidant protection (Murphy, 2012). In the ETC, complexes donate electrons to oxygen producing radicals like superoxide and peroxidases, and high levels of such radicals and oxidative stress cause damage to lipids, enhance DNA breaks, and oxidize nuclear and mtDNA (Tobe, 2013; Czarny et al., 2015). Lower levels of ROS also play a role in normal cellular functioning, such as differentiation of cells, tissue regeneration, redox biology, and promoting adaptation to environmental changes (Vakifahmetoglu-Norberg et al., 2017). The production of highly reactive free radicals is increased when premature leakage of electrons to oxygen occurs in the ETC, increasing oxidative stress. Superoxide is a precursor for ROS, and complex I and III are mainly responsible for its production (Vakifahmetoglu-Norberg et al., 2017). Oxidative stress could be the cause or consequence of damage to mitochondria and mtDNA (Xie et al., 2017). Martins-de-Souza et al. (2012) speculated that a reduction in ATP could be due to oxidative stress and that the increased levels of subunits of OXPHOS complexes were compensatory. In fact, ATP reduction and its relation to oxidative stress have been linked not only to depression (detailed below), but also to psychotic disorders (see Chouinard et al., 2017), autism (Rose et al., 2014), anxiety (Kumar and Chanana, 2017), Alzheimer’s disease (recently reviewed in Tramutola et al., 2017), and Huntington disease (Quintanilla et al., 2017).

Several papers have reported links between oxidative stress and depression. Ben-Shachar and Karry (2008) reported an increase in oxidative damage and alterations in ETC complex I in the prefrontal cortex of depressed patients. Other researchers noted decreased levels of antioxidants and antioxidant enzymes in depression and related these changes to deficits in cognition (Anderson, 2018). In an immobilization stress preclinical model of depression, in which animals were restrained for 6 h a day, levels of the cellular antioxidant glutathione were reduced by 36.7% after 21 days, while lipid peroxidation increased (Madrigal et al., 2001). The authors speculated that lipid peroxidation could cause mitochondrial dysfunction by damaging membranes and causing excitotoxicity, which could be potentiated by increased production of reactive molecules (Braughler and Hall, 1989) or decreased antioxidant levels. In the olfactory bulbectomy model of depression, glutathione levels were also decreased whereas ROS superoxide, nitric oxide (NO), and lipid hydroperoxide levels were increased in mice (Holzmann et al., 2015) and rats (Almeida et al., 2017).

As previously mentioned, the effect of 21 days of environmental stress (i.e., restraint) on mitochondrial dysfunction in rats was investigated (Madrigal et al., 2001). The authors reported that mitochondrial activity of ETC complexes I–III were significantly decreased after just 7 days of restraint stress (6 h per day); however, there was no difference in complex IV and no stress-induced decreases in oxygen consumption throughout the 21-day period. They speculated that mitochondrial dysfunction was a result of overproduction of NO, as an accumulation of NO metabolites was found in the brain tissue. Similarly, 40 days of chronic variable stress decreased sucrose preference as well as inhibited ETC complexes I, II, and IV in the cerebral cortex and cerebellum of rats (Rezin et al., 2008b). Interestingly, increased expression of proteins related to mitochondrial import and transport in the OXPHOS pathway was also seen in a preclinical mouse model of anxiety, a disorder that is highly comorbid with depression (Filiou et al., 2011). Moreover, in this model, the expression of enzymes involved in catalyzing glycolysis pathway reactions was also dysregulated.

The mechanisms by which environmental stress negatively impacts the brain are still not fully understood. However, there is evidence that free-radicals such as NO cause rapid damage to certain cell macromolecules that are involved in the ETC system, which in turn will decrease production of ATP and may be implicated in cytotoxic effects in the central nervous system (Cleeter et al., 1994; Lizasoain et al., 1996). Madrigal et al. (2001) did not find changes in ATP levels, which provides further evidence that a threshold of ETC complex dysfunction may have to be reached before the capability of mitochondria to maintain homeostasis diminishes (Davey and Clark, 1996).

Antidepressant treatment improves oxidative stress parameters in patients with depression. For example, a higher serum total oxidant status and a lower serum total antioxidant capacity in depressed patients were normalized after 42 days of antidepressant treatment (Cumurcu et al., 2009). Similar findings have been reported in animal models of depression. Venlafaxine increased expression of antioxidant mitochondrial genes in the mouse brain, which reduced levels of hydrogen peroxide and peroxynitrite (Goemaere and Knoops, 2012; Tamási et al., 2014). Furthermore, in the chronic mild stress paradigm, lamotrigine, aripiprazole, and escitalopram all normalized glutathione and glutathione peroxidase activity in rat cortical regions (Eren et al., 2007a). Lipid peroxidation in the cortex and plasma was increased by chronic mild stress, but also reversed by the same three treatments. A similar study revealed that venlafaxine can reverse chronic mild stress-induced decreases in glutathione peroxidase activity and vitamin C, and increases in lipid peroxidation and NO in the rat cortex (Eren et al., 2007b). Moreover, unpredictable stress in mice resulted in increased open field test exploration along with decreased liver glutathione, superoxide dismutase, and total antioxidant capability, which was reversed by the traditional Chinese medicine, Shudihuang, in a dose-dependent manner (Zhang et al., 2009).

## Reelin, Oxidative Stress, Inflammation, and Depression

A further link between ROS and depression has been suggested by recent work focused on the extracellular matrix protein reelin. Reelin has been linked to depression in preclinical models of depression: A decline in reelin expression in the hippocampal subgranular zone is associated with the emergence of depression-like behavior, and heterozygous reeler mice (HRM) with 50% of the normal levels of reelin are highly susceptible to the depressogenic effects of stress hormones (Lussier et al., 2011, 2013). Interestingly, a subpopulation of reelin containing cells also coexpress nNOS, and the percentage of neurons coexpressing both markers is specifically decreased in the subgranular zone and molecular layer of the dentate gyrus in HRM (Romay-Tallon et al., 2010).

As reelin secretion by neurons in the subgranular zone may be involved in regulating the maturation of adult hippocampal newborn neurons (Lussier et al., 2009), and as deficits in adult hippocampal neurogenesis have been proposed to be a key event underlying the development of a depressive phenotype (detailed above in section “Hypotheses About the Neurobiological Basis of Depression”), we recently examined the effects of repeated CORT injections on coexpression of reelin and nNOS across hippocampal subregions in brains from HRM and wildtype mice. We found that repeated CORT (administered at a dose that induces depression-like behavior in HRM; Lussier et al., 2011) creates an imbalance between reelin and nNOS expression in the proliferative subgranular zone of the dentate gyrus, with CORT inducing a decrease in colocalization of reelin and nNOS in wildtype mice but a significant increase in colocalization of these markers in HRM. We interpreted these results as being indicative of profound excitotoxicity in dentate gyrus neurons after chronic exposure to stress hormones to a degree that produces depression-like behavior (Romay-Tallon et al., 2010, 2015).

Nitric oxide and other ROS inhibit mitochondrial 2-oxoglutarate dehydrogenase giving rise to increased levels of glutamate, which eventually leads to glutamate excitotoxicity and cell death (Weidinger et al., 2017). The reelin–nNOS connection should receive more experimental attention, as a number of reports indicate that alterations in reelin expression within the dentate gyrus may result in deficient maturation of newborn granule neurons and dampened hippocampal plasticity, and may represent a key event in the pathophysiology of depression (reviewed in Caruncho et al., 2016).

There is also a key link with inflammation to consider in the context of these experiments. Many studies support the idea that inflammatory processes are involved in depression, and in fact targeting of inflammatory cytokines to reduce depression symptoms is a very active area of research (recently reviewed by Shariq et al., 2018). Studies have shown that pro-inflammatory cytokines alter ETC complexes and complex associated enzymes (Samavati et al., 2008), and that they activate pro-apoptotic proteins and the caspase cascade (Bansal and Kuhad, 2016). In mice, the injection of lipopolysaccharide, which induces strong immune responses and secretion of pro-inflammatory cytokines, significantly increased depression-like behavior in the sucrose preference and forced swim tests, and decreased ATP levels and mitochondrial membrane potential in the hippocampus (Chen et al., 2017).

Alterations in several components of the immune system, and in inflammatory markers, have also been observed in animals with low or null reelin expression (Green-Johnson et al., 1995). We have reported that these animals not only are quite susceptible to the depressogenic effects of repeated CORT (Lussier et al., 2011), but also show alterations in the clustering of specific membrane proteins in lymphocytes (Rivera-Baltanás et al., 2010) which prompted us to investigate membrane protein clustering in lymphocytes in depression patients, and to propose that the pattern of clustering of specific proteins along the plasma membrane of lymphocytes could be a putative biomarker of depression, and perhaps underlie some of the inflammatory events observed in depression patients (Rivera-Baltanas et al., 2012, 2014, 2015). In fact, alterations in oxidative stress in lymphocytes have been clearly demonstrated in depression (Szuster-Ciesielska et al., 2008; Czarny et al., 2018). Following up this line of thought, we have recently demonstrated that peripheral injections of the anti-inflammatory drug etanercept (which is unable to cross the blood–brain–barrier) not only rescues the depression-like behavior induced by repeated CORT but also normalizes the neurochemical phenotype of reelin expressing cells in the hippocampal dentate gyrus. We speculated that both peripheral and secondary central actions may be operative in the antidepressant effects of etanercept injections (Brymer et al., 2018). It seems clear that additional studies would be required to determine the connection between reelin, oxidative stress, and inflammation in depression, not only to determine how these factors may be an important component of the pathophysiology of depression, but also to evaluate them as possible targets to develop novel antidepressant drugs.

## Apoptosis

Mitochondria have a clear role in cell metabolism, and evidence suggests that mitochondrial morphology also affects metabolic enzymes through fusion and fission (Chen et al., 2005). The formation and morphology of cristae on the inner membrane, which regulate mitochondrial metabolism, may require fusion machinery as a loss of such machinery results in decreased metabolism (McBride et al., 2006). Abnormal cell structure and function could result in alterations in synaptic signaling and neural circuits and vice versa (Kaidanovich-Beilin et al., 2012). Excessive glutamatergic activation of NMDA receptors was shown to increase ROS levels and alter mitochondrial membrane polarity, which led to elevated apoptosis rates in cardiomyocytes, possibly as a result of increased calcium ion influx (Gao et al., 2007). Mitochondria are present at synapses and responsive to synaptic stimulation (McBride et al., 2006). As the hippocampus is highly vulnerable to the depressogenic effects of chronic stress, it is likely that hippocampal mitochondria behave abnormally in depressed patients. For example, the clustering of mitochondria in dendritic spines in response to neural activity may be altered (Li et al., 2004). Glucocorticoid receptors (GRs) are also highly prevalent in the hippocampus. These receptors are activated when stress hormone levels are high, such as during periods of chronic stress. GRs coordinate OXPHOS enzyme biosynthesis (Simoes et al., 2012) and regulate mitochondrial gene transcription such as cytochrome oxidase 1 and 3, the activity of which correlates with levels of ATP (Adzic et al., 2013). In the hippocampus, chronic stress altered the phosphorylation of mitochondrial GRs, whereas in the prefrontal cortex, chronic stress significantly increased mitochondrial GR levels (Adzic et al., 2013). In the gut, stress increased serum CORT levels, which activated GR recruitment to instigate decreased ETC complex I activity, hyper-fission, and accumulation of ROS inducing apoptosis (De et al., 2017). This is the intrinsic pathway of apoptosis, which is affected by oxidative stress, elevated Ca2+ levels, and damaged DNA (Green and Kroemer, 1998; Kroemer et al., 2007).

Mitochondria are also involved in apoptosis through the extrinsic pathway, in which the death-inducing signaling complex is formed, leading to activation of caspase-8 and then downstream caspases that target substrates leading to programmed cell death (Vakifahmetoglu-Norberg et al., 2017). Other proteins such as K-Ras or BH3 interacting domain death agonist can also induce cell death when activated by caspases by translocating to mitochondria, where they trigger the release of the executioner caspases (Bivona et al., 2006; Bansal and Kuhad, 2016).

It is important to note that the effect of stress on mitochondrial function may depend on the nature of the stressor or period of chronicity. Although chronic stress and high levels of circulating stress hormones are a clear risk factor for depression (Gibbons and McHugh, 1962; Holsboer, 2001; Parker et al., 2003), low levels of stress hormones can be beneficial. For example, the effects of the stress hormone CORT on depression-like behavior in rodent models depend on the dose and time period of administration: higher doses and longer periods of administration produce robust increases in depression-like behavior but low doses or high doses given for short periods do not (Johnson et al., 2006; Lussier et al., 2013). This is consistent with the effects of stress hormones on mitochondria. Glucocorticoids can translocate to mitochondria, where they inhibit the release of cytochrome c and decrease apoptosis (Du et al., 2009). However, this is dependent on the level of glucocorticoids present in the tissue. Du et al. (2009) revealed that low doses of CORT were neuroprotective through regulation of mitochondria, but high doses were neurotoxic. Similarly, inhibiting mitochondrial protein synthesis completely impairs neuronal differentiation, but inhibiting ATP synthetase alone does not affect neurogenesis (Vayssiere et al., 1992). It would be of interest to map the dose-dependent effects of glucocorticoids on markers of mitochondrial function along with depression-like behavior to further confirm these relationships.

## The Effect of Antidepressants on Mitochondria

There has been quite a bit of work done to try to understand the effect of antidepressant drugs on mitochondrial function. Most antidepressants work by increasing synaptic levels of serotonin and/or norepinephrine, and adverse side effects are commonly reported. Much of the research done to examine links between antidepressant drugs and mitochondrial function have used the SSRI fluoxetine, which may either inhibit or trigger mitochondrial apoptosis and alter activity of the ETC, depending on the cell type (de Oliveira, 2016). In the rat liver, fluoxetine administered *in vitro* inhibited state 3 of mitochondrial respiration for α-ketoglutarate and succinate oxidation, stimulated state 4 for succinate, and decreased the respiratory control ratio for both oxidizable substrates (Souza et al., 1994). The same effects were found in a later study on the rat brain, with fluoxetine decreasing the rate of ATP synthesis (Curti et al., 1999), and a study on the pig brain, showing that fluoxetine can inhibit mitochondrial function (Hroudová and Fišar, 2012). These results indicate that high doses of fluoxetine have negative effects on mitochondria. Fluoxetine crosses mitochondrial membranes with ease, and it is possible that fluoxetine could interfere with membrane-bound proteins causing pro-apoptotic events (de Oliveira, 2016).

*In vivo* studies reveal a slightly more complex scenario, in that fluoxetine has both beneficial and detrimental effects on mitochondria when given systemically. After a single injection of fluoxetine (25 mg/kg), activity of the Krebs cycle enzyme citrate synthase was increased in the striatum, but not in the prefrontal cortex or hippocampus, and the striatal increase was no longer evident after 28 days of treatment (Agostinho et al., 2011a). Fluoxetine at the same dose also increased activity of ETC complex I in the hippocampus after one injection, but not in the prefrontal cortex or striatum (Agostinho et al., 2011b). However, after 28 days of daily injections, complex IV activity was decreased in the hippocampus. In another study, 21 days of low dose fluoxetine injections (5 mg/kg) increased expression of cytochrome oxidase 1 and cytochrome oxidase 3 mRNA in the prefrontal cortex in female rats, but not male rats, and decreased cytochrome oxidase 1 and cytochrome oxidase 3 mRNA in the hippocampus of male rats but not female rats (Adzic et al., 2013). These results suggest sex and region specific effects of systemic fluoxetine on mitochondrial function.

There has been some work done to examine the effect of other antidepressants. For example, chronic treatment with the tricyclic antidepressant imipramine as well as electroconvulsive shocks increased levels of cytochrome b mRNA in the rat cortex but not in the hippocampus, cerebellum or liver (Huang et al., 1997). Cytochrome b mRNA translates a protein that is involved in ETC complex III functioning. In addition, the SNRI venlafaxine actually had detrimental effects on complex IV of the ETC, although it increased expression of anti-apoptotic and antioxidant mitochondrial genes (Tamási et al., 2014). Finally, fluoxetine and desipramine enhanced cytochrome oxidase and glutamate dehydrogenase in presynaptic mitochondria located in the rat hippocampus (Villa et al., 2017). These data highlight the importance of antidepressants at a subcellular level and suggest that mitochondrial energy metabolism could be a mechanism of antidepressant drug action.

## Gender Differences in Depression and Mitochondria

Women are more than twice as likely to suffer from depression than men, but it is not yet clear why this occurs and whether or not it has a biological basis. There is evidence that gender differences might arise due to decreased levels of circulating estrogens (Bloch et al., 2003), which is reinforced by observations that ovariectomy increases depression-like behavior in mice subjected to a chronic unpredictable stress paradigm (Lagunas et al., 2010). Furthermore, administering estradiol alleviates depression-like symptoms in ovariectomized rats (Rachman et al., 1998) and may accelerate antidepressant effects in humans (Rasgon et al., 2007). The evidence linking mitochondria to estradiol and depression is sparse, but emerging. Some studies have indicated a protective role of estradiol in mitochondria, showing that it can inhibit the passage of ROS into mitochondria as well as preventing mitochondrial collapse and increasing the rate of ATP synthesis (Wang et al., 2003; Shimamoto and Rappeneau, 2017). Mitochondria are known to express estrogen and GRs in lung tissue, suggesting that mitochondria are responsive to fluctuating levels of stress hormones and estradiol (Walf and Frye, 2006). It seems that mitochondrial estrogen and GRs in lung tissue are involved in the biosynthesis of OXPHOS enzymes, which will affect other mitochondrial functions such as apoptosis and ROS production (Simoes et al., 2012). It would be quite interesting to follow these studies with an investigation of brain mitochondria and estrogen receptors to determine whether sex steroid hormones in the brain might be involved in the gender differences seen in the prevalence of depression.

## Conclusion

The specific biological mechanisms underlying major depression have yet to be elucidated. This review highlights the potential importance of mitochondrial function in depression. This is an area that has received relatively little experimental attention, but the data that have been published to date are promising and should be pursued. Although one must be cautious in extrapolating findings from preclinical animal models to the human condition, there is evidence that chronic stress-induced inhibition of ETC complexes in the inner membrane of mitochondria is a contributing factor in the pathophysiology of depression. Dysfunctional mitochondria decrease the pool of available ATP, which could have detrimental effects on signal transduction pathways, dampening activity in neuronal circuits, and interfering with mitochondrial fusion and fission. This negative cascade would ultimately increase oxidative stress, inflammatory responses, and pro-apoptotic events, some of which are known to be involved in the pathogenesis of depression. Viewed this way, it seems logical that reversing the early stages of mitochondrial dysfunction could provide a novel target for therapeutic intervention.

## Author Contributions

All authors contributed to the writing of this manuscript. JA wrote the first draft. RR-T, KB, HC, and LK edited the draft. JA constructed the figure. LK finalized the manuscript.

## Conflict of Interest Statement

The authors declare that the research was conducted in the absence of any commercial or financial relationships that could be construed as a potential conflict of interest.
